# Experiences of using the Recommended Summary Plan for Emergency Care and Treatment (ReSPECT) in English general practice: a qualitative study among key primary health and social care professionals, patients, and their relatives

**DOI:** 10.3399/BJGP.2024.0248

**Published:** 2025-01-14

**Authors:** Anne-Marie Slowther, Celia Janine Bernstein, Caroline Huxley, Jenny Harlock, Karin Eli, Claire Mann, Rachel Spencer, Jeremy Dale, Paramjit Gill, Hazel Blanchard, Martin Underwood, Frances Griffiths

**Affiliations:** Warwick Medical School, University of Warwick, Coventry.; Warwick Medical School, University of Warwick, Coventry.; Warwick Medical School, University of Warwick, Coventry.; Warwick Medical School, University of Warwick, Coventry.; Warwick Medical School, University of Warwick, Coventry.; Warwick Medical School, University of Warwick, Coventry.; Warwick Medical School, University of Warwick, Coventry.; Warwick Medical School, University of Warwick, Coventry.; Warwick Medical School, University of Warwick, Coventry.; Forrest Medical Centre, Coventry.; Warwick Medical School, University of Warwick, Coventry.; Warwick Medical School, University of Warwick, Coventry.

**Keywords:** emergency care treatment plans, general practice, patient-centred care, qualitative research, ReSPECT

## Abstract

**Background:**

The Recommended Summary Plan for Emergency Care and Treatment (ReSPECT) has been implemented in many areas of the UK. It is unclear how ReSPECT is used in primary and community care settings.

**Aim:**

To investigate how the ReSPECT process is understood and experienced in the community by clinicians, social care staff, patients, their relatives, and identify obstacles and enablers to its implementation.

**Design and setting:**

A qualitative interview and focus-group study across 13 general practices in three areas of England, between January and December 2022.

**Method:**

We interviewed GPs, specialist nurses, patients and relatives, and senior care home staff. Focus groups were conducted with community nurses, paramedics, and home care workers. Questions focused on understanding experiences of, and engagement with, ReSPECT. We analysed data using thematic analysis and a coding framework drawn from normalisation process theory.

**Results:**

Participants included *n* = 21 GPs, *n* = 5 specialist nurses, *n* = 9 patients, *n* = 7 relatives, *n* = 31 care home staff, *n* = 9 community nurses, *n* = 7 home care workers, and *n* = 2 paramedics. Participants supported ReSPECT, regarding it as a tool to facilitate person-centred care. GPs faced challenges in timing the introduction of ReSPECT and ensuring sufficient time to complete plans with patients. ReSPECT conversations worked best when there was a trusting relationship between the clinician and the patient (and their family). Anticipating future illness trajectories was difficult, yet plans were rarely reviewed. Interpreting recommendations in emergencies was challenging.

**Conclusion:**

The ReSPECT process has not translated as well as expected in the community setting. A revised approach is needed to address the challenges of implementation in this context.

## Introduction

The Recommended Summary Plan for Emergency Care and Treatment (ReSPECT) is an emergency care and treatment planning (ECTP) process launched by the Resuscitation Council UK (RCUK) in 2016.^1^ Initially introduced in acute NHS trusts it is now used in nearly half of general practices in England.^2^ The ReSPECT process involves discussion between a patient (or the family of someone who lacks capacity) and a healthcare professional about the patient’s preferences for treatment in the event of an emergency or an acute deterioration in their condition. A ReSPECT plan records the patient’s values and preferences, and agreed specific clinical recommendations about future emergency treatment including cardiopulmonary resuscitation (CPR).^3^ The Care Quality Commission’s report on use of ‘do not attempt cardiopulmonary resuscitation’ (DNACPR) recommendations during the COVID-19 pandemic cited ReSPECT as an example of good practice in enabling these conversations.^4^

An evaluation of the ReSPECT process in early-adopter acute NHS trusts before COVID-19 found that hospital clinicians engaged with the process but many felt that GPs were best placed to have these conversations.^5^ A linked focus-group study with GPs found that they conceptualised ReSPECT as an end-of-life planning process, using it to plan with patients for a dignified, pain-free death.^6^ The GPs found recommendations on hospital-completed forms were not always useful in a community setting. An interview study with GPs and care home staff in the west of England found a generally positive attitude to the use of ReSPECT, but noted its use was complex and identified challenges in incorporating patients’ preferences into decision making.^7^ A survey of GPs in England found broad support for a range of community-based health and social care professionals completing ECTPs.^2^

There is limited evidence on how ReSPECT is currently used in primary and community care settings. To address this knowledge gap we conducted a qualitative exploration of the use of ReSPECT, once embedded in general practice and after the initial COVID pandemic. We investigated how ReSPECT was understood and experienced by GPs, other health and social care professionals who work with GPs, patients, and/or their relatives, identifying obstacles and enablers to its implementation. The study was part of a large mixed-methods study on the use of ReSPECT in primary care in England.^8^

## Method

We interviewed GPs and specialist nurses working in general practice and visited their practices to speak with clinical and administrative staff about how they engaged with the ReSPECT process. We interviewed patients with a ReSPECT plan and/or their relatives about their experience of the process. We interviewed managers and senior carers in care homes associated with the study GP practices and ran focus groups with community-based nursing staff, paramedics, and home care workers to capture their views. Our study team comprised six clinical academics, a behavioural scientist, and four social scientists. A lay advisory group contributed to the study development and delivery at each stage. A detailed description of the study methods is reported elsewhere.^8^

### Recruitment

Three clinical research networks (CRNs) recruited 13 practices across three diverse clinical commissioning group (CCG) areas in England. Information about the study was sent to research-active practices and expressions of interest sought. Of these practices, 14 expressed an interest but one practice did not respond to follow-up emails. In each participating practice, two/three GPs and/or specialist nurses in the practice, who were involved in completing ReSPECT plans with patients, were invited to take part in an interview. During one-day visits to practices the researcher invited available staff members to take part in a brief conversation about how ReSPECT worked in the practice. Practice staff received information about the study before the visit.

**Figure 1.**
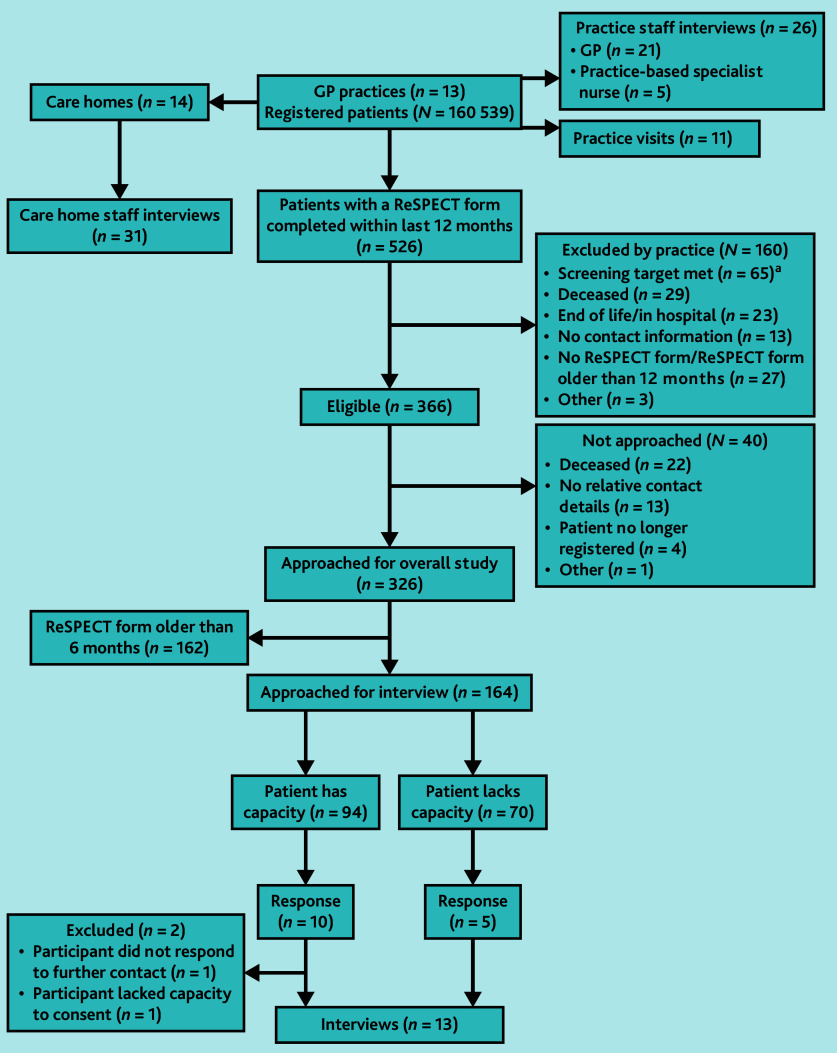
Recruitment flow chart for interview study. ^a^ Target sample was *n* = 40 patients in each practice Two practices exceeded this number for patients with a ReSPECT plan in previous 12 months so *n* = 40 were randomly selected for sample. ReSPECT = Recommended Summary Plan for Emergency Care and Treatment.

The practice sent an invitation to take part in an interview to patients who had a record of a completed ReSPECT plan in the previous 6 months. If the patient was known to lack capacity, their designated next of kin was approached. In participating care homes, we purposively sampled staff most likely to have engaged with the ReSPECT process (care home managers or their deputy, senior carers, and care coordinators) (see Figure 1 for interview recruitment process).

We advertised the study through local community nursing networks, practitioner teams, and private home care organisations to recruit for focus groups.

### Data collection

Interview and focus-group participants received an information sheet and consent form to read in advance. Participants were given the choice of remote (telephone, Microsoft Teams™, or Zoom™) or face-to-face interviews. Verbal consent was obtained for remote interviews and written consent for face-to-face interviews. Interviews were audio-and/or video-recorded, apart from one GP and one care-home group interview where the participants did not consent to this. Detailed field notes were documented with consent instead. During visits to GP practices, anonymised verbal consent was obtained and recorded immediately before conversations with clinical and administrative staff. Anonymised field notes of conversations were taken.

Data collection occurred between January and December 2022. Topic guides for interviews, focus groups, and conversations with practice staff were informed by the research questions and further developed with contributions from the study team and lay advisory group. GP, care home staff, and community staff topic guides explored participants’ experiences of implementing the ReSPECT process in the context of their work. Guides for patient and relative interviews explored their experience of completing a ReSPECT plan and their understanding of its purpose (Supplementary Boxes S1–S6). Interviews, focus groups, and practice visits were carried out by six early to mid-career post-doctoral researchers (a sociologist, social and policy scientist, research psychologist, medical anthropologist, health services researcher, and education researcher, who are all authors of this article). All six were experienced in qualitative methods. Audio-recorded data were transcribed by professional transcribers. Recordings and transcripts were anonymised. Data collection continued until no new relevant knowledge was obtained.^9^

### Data analysis

An a priori coding framework with key analysis questions using the main constructs of normalisation process theory (NPT) (coherence building, cognitive participation, collective action, and reflexive monitoring) was developed for all health and social care staff data (Box 1).^10^ During analysis meetings, seven study team members developed and refined the key analysis questions to increase the relevance for the dataset. The framework was used to code transcripts of all focus group interviews and 50% of interviews with GPs and care home staff, purposively sampled to capture all locations and staff roles. The coding was completed in NVivo software (version 1.6.1 QSR). Memos documented emergent themes and key messages, while field notes supplemented interview data. Members of the study team read through and documented any new codes or nuances in the remaining data.^11^

**Box 1.** Normalisation process theory (NPT) coding framework

**NPT construct (May *etal*, 2013)^10^**	**Process (Lund *et al*, 2015)^14^**	**Key questions**
**Coherence building**	Participants attribute meaning to the activities that surround a practice and make sense of its possibilities within their field of agency	What do GPs, specialist nurses, admin staff, care home staff, and community health and social care professionals (H&SCP) understand as the meaning of ReSPECT within the context of their role?

**Cognitive participation**	Participants legitimise and enrol themselves and others into activities around a practice	Who initiates the ReSPECT process in the practice or care home setting, and how and when?
		Who initiates the ReSPECT process with patients, and how and when?
		How do clinicians, care home staff, and community H&SCPs respond to and engage with the ReSPECT process? (Preparation/having the conversation/using the form in an emergency)
		What do clinicians, care home staff, and community H&SCPs think about the value of ReSPECT to the health service?
		What do clinicians, care home staff, and community H&SCPs think about the value of ReSPECT for individual patients?

**Collective action**	Participants mobilise skills and resources needed to enact a practice	Are there any barriers to cooperation or communication between different stakeholders? What workarounds have practices and care homes developed in their implementation of ReSPECT?
		What elements of ReSPECT are unable to fit into working practice?
		What do clinicians need to become skilled at holding ReSPECT conversations and writing recommendations?

**Reflexive monitoring**	Participants assemble and appraise information about the effects of a practice and utilise that knowledge to reconfigure relationships and behaviours	What feedback do practices and care homes seek/get on how ReSPECT is operating within their setting?
		What processes do practices have in place for reviewing ReSPECT plans?
		What informal feedback on the ReSPECT process do clinicians and care home staff receive from patients or colleagues? For example, on their experience of the process, on their understanding of the form, or to check whether recommendations are still relevant
		Has implementation of ReSPECT changed because of feedback from patients or other stakeholders? For example, change after GPs reflected on practice

**Table:** How this fits in

The Recommended Summary Plan for Emergency Care and Treatment (ReSPECT) is a specific model of emergency care treatment planning now used in primary care and hospitals, and in many areas of the UK. It has been evaluated in hospital settings, but little is known about how it is understood and operationalised in general practice. Our research found a consensus that ReSPECT could facilitate a person-centred approach to future treatment decision making, but there are specific challenges in implementing ReSPECT in a community setting. A revised approach needs to consider uncertainty of illness trajectories over time and to emphasise patient values to facilitate decision making in an emergency.

An inductive thematic analysis was adopted for patient and relative interviews.^12^^,^^13^ The fifth author coded all transcripts to capture meanings and identified candidate themes. The third and fourth authors double-coded eight interviews (62%) and consensus was reached on themes in analysis meeting discussion. We then compared findings across all datasets to provide an in-depth picture of how ReSPECT is used in general practice, including facilitators and obstacles to implementation.

In this article we focus on the experience of creating and implementing ReSPECT plans in primary care, identifying themes that cut across the NPT and thematic analyses.

## Results

The study recruited 13 GP practices situated predominantly in urban/suburban areas. Overall, they were nationally representative in terms of deprivation. Minority ethnic groups appeared to be slightly underrepresented, with 89% of patients being recorded as white (Table 1). We interviewed *n* = 21 GPs, *n* = 5 specialist nurses, and *n* = 31 senior care home staff (care home managers or their deputies *n* = 12, senior care coordinators *n* = 16, and nursing leads *n* = 3). We interviewed *n* = 6 patients, *n* = 4 relatives of patients with a ReSPECT form, and *n* = 3 patient/relative pairs. This corresponded to 13 patients with a ReSPECT form (*n* = 8 women and *n* = 5 men aged 53–93 years).

**Table 1.** Characteristics of patients registered with participating practices (*n* = 13 practices), total patients registered *n* = 162 798^a^

**Characteristic**	**Patients registered with participating practices, *n* (%)**	**Population in England, %**
**Sex**		
Male	81 399 (50.0)	50.0
Female	81 399 (50.0)	50.0
**Age, years**		
<20	36 354 (22.3)	23.1
20–59	84 592 (52.0)	52.6
60–79	33 443 (20.5)	19.3
≥80	8409 (5.2)	5.0
**Ethnicity^b^**		
White	145 387 (89.3)	81.7
Asian	11 645 (7.2)	—
Black	2430 (1.5)	—
Mixed	2162 (1.3)	—
Non-white ethnic group	619 (0.4)	—
Other	555 (0.3)	—
**Deprivation, decile^c^**	**Number of practices in each decile, *n* (%))**	**Registered patients across all study practices in each decile, %**
1	1 (8)	5
2	2 (15)	12
3	1 (8)	9
4	2 (15)	5
5	2 (15)	16
6	0	0
7	2 (15)	16
8	2 (15)	28
10	1 (8)	7

a *Number of registered patients differs from that in Figure 1, reflecting different dates of collection.*

b *Ethnicity as reported in practice-level data may not match census categories, and recording may be incomplete. Therefore, only the national percentage for white ethnic category (Census data 2021) is provided.^15^*

c *Number of practices in each decile and percentage of total population registered with a practice in each decile band. Lower decile indicates more deprived All practice data obtained from Public Health England.^16^*

One-day fieldwork visits took place at 11 GP practices (we were unable to arrange visits at two practices). Informal conversations were carried out with clinical (*n* = 49) and administrative (*n* = 75) staff within their work setting. Focus-group participants included, *n* = 9 community nurses, *n* = 2 paramedics, and *n* = 7 home care workers. See Table 2 for summary of interview types.

**Table 2.** Participant numbers for type and location of interview

**Participant**	**Individual interview, *n* = 33**	**Group/paired interview, *n* = 16**	**Focus group, *n* = 20**	**Remote (via Teams™), *n* = 67**	**Face to face, *n* = 23**	**Location if face to face**
**Practice staff (*N* = 26)**						
GP	21		0	20	1	Practice
Practice-based specialist nurse	5		0	5	0	

**Care home staff**						
Care home manager	10		2	5	7	Care home
Care coordinator/senior carer	6	10 (2, 3, 5)^a^		7	9	Care home
Senior nurse/nursing lead	3		0	2	1	Care home

**Other health and social care professionals**						
Community nurse			9	9		
Paramedic			2	2		
Home care worker			7	6		

**Patients and relatives**						
Patient	6	3		6 (telephone)	3	Patient’s home
Relative	4	3		5 (telephone)	2	Relative’s home

a *Numbers of participants in each group/paired interview.*

Our findings are presented in four themes that frame how ReSPECT is understood and operationalised in general practice. Our final themes were situated within the underlying theoretical framework of NPT: coherence building (conceptualising the ReSPECT process), cognitive participation (ReSPECT requires time, timing and preparation), collective action (importance of relationships, and negotiating uncertainty, complexity, and responsibility), and reflexive monitoring (captured in the theme of negotiating uncertainty, complexity, and responsibility).

### Conceptualising the ReSPECT process

All participants viewed ReSPECT as a mechanism for facilitating person-centred care, empowering patients/residents to express their wishes and engage in decisions about their future care. Generally, there was agreement that ReSPECT was *‘much wider than just a DNACPR* f*orm’* (GP1), though some patients and GPs spoke only about the CPR recommendations. GPs and nurses described ReSPECT as providing a prompt for conversations with patients about what mattered to them:

*‘The ReSPECT form … helps you to know these patients well … they’re losing all their control over everything in their lives so it’s just listening really and making the time to do that.’*

(Specialist nurse 2)

Care home and home care staff emphasised the role of ReSPECT in making the person’s wishes known:

*‘With the ReSPECT form, we can respect their wishes: We’ve got that document to reassure us that we’re doing the right thing.’*

(Care home manager/Deputy 3)

Several participants felt the ReSPECT conversation provided reassurance for people who feared that a DNACPR recommendation would lead to them not receiving appropriate care:

*‘… I think the ReSPECT process has opened the door to have a freer conversation with people …* [they] *can say, “Well actually, I don’t want to be resuscitated but if I did get an infection that could be treated then I would want that.”’*

(Care home manager/Deputy 1)

Practice staff saw ReSPECT as a process primarily for people who were older and frail, receiving palliative care or in the late stages of a chronic illness. This understanding of ReSPECT was reflected in the systems they used for prompting initiation of ReSPECT conversations, namely inclusion in the palliative care register or admission to a care or nursing home. Patients and relatives mainly described their experience of the ReSPECT process as focusing on the end of life. However, GPs also described conversations about other scenarios:

*‘So where infections are not responding to community treatments or when say there’s an injury like a hip fracture that would need fixing in order to provide pain relief, then obviously we can’t do that in the community, we’d need to admit him to hospital for that to happen.’*

(GP20)

GPs and specialist nurses felt that, for ReSPECT to be fully realised as a means to empower patients, conversations should occur, whenever possible, while the person still has capacity. Delaying conversations until later in the disease trajectory could devalue this central objective:

*‘… it all gets left a little bit to the last minute and we’re writing these in a much more emotive time when they’re, lot more unwell, or they’re not able to actually be involved in the decision.’*

(GP18)

Some GPs and care home staff conceptualised ReSPECT as part of a suite of plans that interact to support holistic care and envisaged them being used with other care plans, even in an emergency:

*‘So, most things asked in a ReSPECT form, I try to mention the important things. The rest, I just write there, “Please follow the care plan.”’*

(GP5)

### ReSPECT requires time, timing, and preparation

GPs and specialist nurses discussed the importance of giving enough time for ReSPECT conversations and investing time in preparing the patient and their family for plan completion. Many plans were initiated following a life-changing diagnosis or deterioration in condition, which required a balance between the need to ascertain a patient’s wishes for future care and allowing them time to come to terms with their new situation:

*‘… there have been a few situations where we’ve spoken to patients who have had bad news that their cancer has progressed … I have sort of tested the water and said, “It, it’s important that whilst you’re still relatively well that we have a discussion about future care … and it’s been quite, quite clear when they have not felt ready to, so you just leave it and revisit it.”’*

(GP2)

Patients appreciated healthcare professionals taking time to find out what was important to them, to have a *‘proper two-way conversation’* (Patient [P] 5). They also valued being given time to think about when they wanted to have the conversation. One patient described how the idea of ReSPECT was introduced over time and at the patient’s own pace:

*‘So it was gently introduced and left in the air … we’ve been talking for a while about my disease and she* [Macmillan nurse] *did say that it would be good to put a ReSPECT form in place. I said, “I don’t, just don’t feel like doing it at the moment.”* [She] *said, “Oh, right. That’s fine.”’*

(P12)

The importance of preparing someone for a ReSPECT conversation by introducing the concept at an early stage and allaying fears and concerns over time was also noted by some home care workers and care home staff who saw themselves as having a role in this process:

*‘… so, I can sit down long before the doctor has that conversation and introduce* [ReSPECT] *… that’s an important part of what we do, to take away the stigma … that then in turn makes the job a lot easier for the doctor.’*

(Care home manager/Deputy 1)

However, many GPs noted that finding the time for conversations was sometimes a challenge in a busy general practice:

*‘It’s a sort of balance between trying to give the patient the time and space they need to discuss these very important issues with the awareness that there’s other patients that are waiting and, and you’re needing to get back to the surgery*.*’*

(GP17)

### Importance of relationships

ReSPECT conversations worked better when there was an established relationship with the patient. Patients and relatives felt more comfortable speaking to someone whom they knew, and who knew them:

*‘I think if it had been a doctor I didn’t know very well then it would have been much more difficult.’*

(Relative 9)

Knowing the patient well helped GPs put the conversation in a context relevant for that patient:

*‘Because I think it’s important that you know the patient well to be able to have that conversation. You need to know their past medical history and put the ReSPECT form discussion in context … it’s a lot more meaningful to do it that way and it feels more holistic.’*

(GP2)

Several participants across our datasets noted that GPs were not necessarily the best placed healthcare professional (HCP) to have a ReSPECT conversation, particularly when the patient had more contact with other teams, such as palliative care or community nursing:

*‘… whether it’s specialist palliative care nurse or community matron or a district nurse that’s got that relationship, sometimes they’re the better people, I think, than the GP in those situations, because of that closer relationship over a period of time.’*

(Community nurse, Focus group 3)

Care home staff observed that the specialist nurses attached to GP practices, who saw their residents regularly, were well placed to have these sensitive conversations:

*‘… they know our residents … and I think that, if you have an understanding, you know … the residents that you’re talking about, I think it, it makes it a lot easier for those people.’*

(Care home manager/Deputy 6)

GPs and specialist nurses also noted the importance of establishing rapport and engaging with the patient’s family, even if the patient had capacity. This was seen as important to ensure they understood the patient’s wishes and could support the recommendations in an emergency:

*‘… I think it’s important for relatives to feel that they’re involved … so in the event that a patient doesn’t have the ability to make their wishes known … people can, you know, advocate on their behalf. That feels important.’*

(GP1)

Patients also described the importance of family involvement in discussions, both to ensure that the family would know their wishes and advocate for them in an emergency, and as a means of alleviating the uncertainty and burden of future decision making:

*‘… because I can give a copy to my daughter. And I think it puts her mind at rest, puts my mind at rest because I know what I want will be done.’*

(P5)

### Negotiating uncertainty, complexity, and responsibility

GPs and nurses completing ReSPECT plans reflected that it was often challenging to ensure the plan truly captured likely future scenarios and options for treatment, particularly given that recommendations may include specialist hospital as well as community-based treatments. This was one reason many GPs preferred to delay ReSPECT conversations until late in a person’s illness trajectory when future deterioration could be more specifically anticipated. Given the complexity of some patients’ conditions, writing recommendations for every eventuality, even when a patient is nearing end-of-life, could mean that the list *‘would be endless’* (researcher field notes, Area 2) and be difficult for paramedics to read through in an emergency. GPs talked about addressing uncertainty by including a level of *‘sensible vagueness’* (GP1) in the recommendations. Conversely, some participants, in both general practice and care homes, suggested a checklist approach might be useful:

*‘It would be helpful to have maybe more … prompts “Is this patient suitable for ITU* [intensive care unit]*, yes or no?”, “Is this patient suitable for renal dialysis, yes or no?”, all the questions that you would want to have on there to be explicit about the level of care*.*”’*

(Specialist nurse 3)

Communicating uncertainty around illness trajectory and future treatment options, then capturing preferences on the plan, was also a challenge, particularly if the patient was currently stable. As one GP noted, *‘… trying to cover every eventuality is actually quite difficult sometimes when people are coming at it from a very-well perspective’* (GP12). The difficulty of capturing views on future treatment choices at a single moment in time was clearly articulated in the following patient comment:

*‘“Were you to suffer a collapse, would you want acute medical intervention? Or would you want comfort measures only?” That might feel like a yes, no question. Actually, it’s quite a nuanced question in many cases, where I might say it depends on the reason for the collapse. It depends on what acute intervention might do for me … And this is likely to change over the course of the next six months as I get my financial situation in hand and under control.’*

(P3)

Participants across the datasets thought that for this reason review of ReSPECT plans was important. Some GPs described reviewing a plan when a patient’s condition deteriorated; however, only one practice systematically reviewed ReSPECT plans, and then only for patients with dementia. Several GPs cited lack of time to enable reviews. Some care homes prompted reviews of their residents’ ReSPECT plans; however, some patients and relatives were unaware that plans could be reviewed.

The challenge of providing clear, relevant recommendations for potential future scenarios was reflected in participants’ experiences of using the plan to guide decision making in an emergency. Care home staff often found the recommendations vague, or that more specific recommendations did not reflect the actual situation they were facing at the time. This could lead to conflict with attending clinicians:

*‘And we have had a bit of resistance from paramedics saying no, their form says not for hospital admission. But if that person needs to go into hospital to be hydrated because they’re dehydrated, or to have intravenous antibiotics, that’s where we get a little bit of resistance.’*

(Care home manager/Deputy 2)

Community nurses also commented on vagueness in recommendations, particularly when hospital-completed plans recommended ‘ward-based care’:

‘*So, you’re then faced with a ReSPECT form in the community that actually is irrelevant, because what, what does that actually mean?’*

(Community nurse, Focus group 2)

Acknowledging these challenges and the complex nature of decision making, the community GPs and specialist nurses noted that ReSPECT recommendations may not always be followed. Many emphasised that responsibility for the decision rested with the attending clinician and that sometimes it was necessary to make decisions that are *‘against the ReSPECT form or against their* [patient/family] *wishes’* (GP10). The ReSPECT plan was often seen as a guide rather than definitive recommendations to be followed, as described by this specialist nurse:

*‘So, for me, I would always treat within the boundaries of respecting what we’ve got on the ReSPECT form, but then having a discussion … Just so everybody’s on board.’*

(Specialist nurse 1)

However, some patients and GPs viewed ReSPECT recommendations as more determinative, regarding it as a document that *‘tells what action needs to be done … so your* [the patient] *decision is the future if you become really unwell’*

(GP5).

While GPs and specialist nurses were usually clear in how they viewed the authority of ReSPECT recommendations, though with variable perspectives, care home staff expressed uncertainty about whether they must always be followed:

*‘Sometimes, if you feel that the resident needs to go to hospital … because, in case, you never know, they might get better or not … I’m not sure if we can override* their [the resident’s ReSPECT] *decision … without talking to them* [their family].*’*

(Care coordinator/Senior carer 9)

Given the challenges in completing and implementing ReSPECT plans in general practice, participants across all datasets noted the importance of experience and identified a need for training to ensure that the ReSPECT process achieved its aims:

*‘There is no teaching for GP trainees on how to do a ReSPECT form. We have training modules on how to initiate an HRT* [hormone replacement therapy] *conversation, how to initiate a smoking conversation, but there is no training for this empathetic* [conversation]*.’*

(GP7)

*‘So I think with* [community nursing], *they haven’t had any updates or any new training to support their skills and knowledge regarding ReSPECT … they haven’t got the skills, the knowledge or the confidence, and they don’t now see it as their role.’*

(Community nurse, Focus group 3)

## Discussion

### Summary

All participants expressed support for ReSPECT and saw it as a tool to facilitate person-centred care. ReSPECT was primarily seen as something to initiate towards the end of a patient’s life, and this was reflected in the timing and trigger systems for starting a ReSPECT conversation. GPs described challenges in both timing the introduction of a ReSPECT conversation and in ensuring they devoted sufficient time for conversations and to review plans. We found that care home staff have a role in preparing residents and their families for ReSPECT conversations, which can facilitate the process. Participants across the datasets agreed that ReSPECT conversations worked best when the clinician–patient relationship is a trusting one, and engaging the patient’s family in conversations was seen as important in ensuring that patient wishes and recommendations are acted on. A key challenge reported was negotiating the uncertainty inherent in anticipating future illness trajectories and communicating this to patients and their families when eliciting treatment preferences. This can lead to vagueness in recommendations creating further uncertainty when these recommendations are interpreted in an emergency setting. We found variation in the extent to which ReSPECT recommendations were seen as determinative.

### Strengths and limitations

A strength of the study was the different lenses through which the ReSPECT process was explored, including that of patients and relatives. Our large study team brought multiple perspectives to the data, but this required attention to consistency and independent checking of analysis throughout.

A key limitation was that participant recruitment was challenging. Our sample of participating practices had more white British patients than the overall population (though ethnicity data were variably recorded). As such, the study might not have captured views and experiences of the ReSPECT process held by minority ethnic groups. While we recruited our target number of care home staff, we did not achieve the spread across care homes we had aimed for. The study ran during the latter part of the COVID pandemic, which likely impacted GP practice and care home capacity to engage with research.

### Comparison with existing literature

A key requirement for successful implementation of a new healthcare intervention is that staff have a shared understanding of the nature and purpose of the intervention, how it differs from current practices, and that staff value the intervention.^10^ We found consensus among general practice and care home staff, patients, and their relatives that ReSPECT was a valuable tool for involving and empowering patients to express their views and influence future treatment decisions. We found similar views in our recent survey of GPs in England.^2^ The association of ReSPECT with end-of-life care may have a positive effect on its implementation in general practice. Prompts to initiate a conversation can be incorporated into existing systems to identifying relevant patients (palliative care registers or care home admission) and ReSPECT conversations can be linked to other care-planning conversations. However, this narrow focus can mean that others who would benefit from, or wish to have, a ReSPECT plan may be excluded. General Medical Council guidance describes patients approaching the end of their life as those who are likely to die within the next 12 months.^17^ A recent report from the Parliamentary and Health Care Ombudsman’s office on DNACPR conversations noted the risk of discrimination or reinforcing stereotypes in linking these conversations to specific groups or settings.^18^

Challenges in timing a ReSPECT conversation relate in part to its association with end-of-life care, and thus anticipated distress for patients in discussing death and dying. A similar barrier has been noted in advance care planning (ACP) conversations.^19^^–^^22^ We also identified this concern in our study on ReSPECT use in acute NHS Trusts.^23^ This may be related to a perception of a societal reluctance to talk about death and dying, though recent research by Wilson *et al* challenges this perception.^24^ Strategies to mitigate this include conducting preparatory conversations with patients to explore their readiness, recognising that conversations take time and may be multiple, and maintaining a trusting relationship with the patient. However, as our GP participants reported, workload pressure can make it difficult to foster the environment needed for these conversations. The impact of GP workload and organisational changes on GP–patient relationships and continuity of care has been noted in a report by the Royal College of General Practitioners.^25^ The report noted the importance of multidisciplinary teams enabling different professionals to provide whole-person care. Including other health and care professionals in the ReSPECT process may address some of the concerns noted in our study. Responders in our national survey of GPs supported the involvement of other health and care professionals in ReSPECT-plan completion, as did GP participants in a qualitative study by Kesten.^2^^,^^7^ The 2024 UK Ombudsman report specifically recommends that *‘NHS England and ICBs* [Integrated Care Boards] *should expand the number and type of staff who can formally support DNACPR conversations in multiple settings.’*^18^

Uncertainty in predicting illness trajectories and future acute deterioration is a key challenge for both ECTP and ACP implementation in a range of settings.^14^^,^^26^^–^^28^ We have previously found that clinical uncertainty influences secondary care clinicians’ decisions to initiate or complete a ReSPECT plan with a patient in their care.^23^ However, the scope of uncertainty is greater in primary care where the range of potential future clinical events and relevant interventions is not constrained by the nature of an admitting event. An additional challenge for ReSPECT and other ECTPs implemented in a community setting is the time between treatment recommendations being documented and when they may be used for decision making, during which the patient’s clinical, personal, or social context may have changed. GPs’ implicit response to this uncertainty is to initiate ReSPECT conversations when patients are nearing the end of their life when there may be less uncertainty about illness trajectory. They may also manage the uncertainty by providing broad rather than specific recommendations, which can in turn create uncertainty for HCPs who are required to interpret them, often in an out-of-hours setting. Uncertainty in interpreting ReSPECT recommendations has also been noted by Kesten.^7^ Challenges with validity and interpretation of recommendations have been noted with other models of ECTP. In the US, the Physician Orders for Life Sustaining Treatment (POLST) is used extensively. Several studies have noted discordance between POLST recommendations and patient wishes, and others have found there is confusion in interpretation of recommendations in the pre-hospital and emergency department setting.^29^^–^^31^ Clear communication about, and sharing of, uncertainty between professionals, patients, and their families may mitigate some of these challenges, and this should be considered in future training. The need for training in knowledge and skills for successful implementation of emergency care treatment plans has previously been noted by Cummings *et al*.^32^

### Implications for practice

ReSPECT has been embraced by general practice and care home staff as a positive step in prompting and facilitating conversations with patients about care goals and future treatment decisions; however, there are challenges in ensuring that these conversations are timely and meaningful. The inclusion of other HCPs, who may have a more established relationship with the patient, helps mitigate these issues. Perhaps a greater challenge is in agreeing, communicating, and interpreting recommendations in the context of a dynamic, complex web of uncertainty. This calls for transparency and honest communication around the aims, scope, and limits of ReSPECT, or other ECTPs, between professionals, patients, and their families. As ReSPECT plans become embedded in practice, attention is needed on how best to initiate plan reviews, whether and how this is systematised, and increased sensitivity to the need for opportunistic review when required. Training is important when implementing ECTPs to ensure shared understanding and to develop the necessary skills. This training needs to extend beyond GPs to care home staff and other HCPs.

However, training may not in itself address the core issue of how ReSPECT is framed for use in a community setting and the difficulties with its translation into primary care as identified in this study. This is because it does not adequately account for greater clinical uncertainty and a community context. A revised approach, framed for use in a community setting, appears necessary to ensure that patients’ preferences and values are at the forefront of emergency care decision making.

## Data

All data requests should be submitted to the corresponding author for consideration. Access to anonymised data may be granted following review.
